# Crystal structure of *N*-[3-(2-chloro­benzo­yl)-5-ethyl­thio­phen-2-yl]-2-[(*E*)-(2-hy­droxy­benzyl­idene)amino]­acetamide

**DOI:** 10.1107/S1600536814018224

**Published:** 2014-08-16

**Authors:** Manpreet Kaur, Jerry P. Jasinski, Channappa N. Kavitha, Hemmige S. Yathirajan, K. Byrappa

**Affiliations:** a Department of Studies in Chemistry, University of Mysore, Manasagangotri, Mysore 570 006, India; b Department of Chemistry, Keene State College, 229 Main Street, Keene, NH 03435-2001, USA; c Materials Science Center, University of Mysore, Vijyana Bhavan Building, Manasagangothri, Mysore 570 006, India

**Keywords:** crystal structure, thio­phene derivatives, Schiff bases, hydrogen bonding

## Abstract

In the title compound, C_22_H_19_ClN_2_O_3_S, the dihedral angle between the mean planes of the thio­phene ring and the chloro­phenyl and hy­droxy­phenyl rings are 70.1 (1) and 40.2 (4)°, respectively. The benzene rings are twisted with respect to each other by 88.9 (3)°. The imine bond lies in an *E* conformation. Intra­molecular O—H⋯N and N—H⋯O hydrogen bonds each generate *S*(6) ring motifs. In the crystal, weak C—H⋯O inter­actions link the mol­ecules, forming chains along the *c* axis and zigzag chains along the *b* axis, generating sheets lying parallel to (100).

## Related literature   

For background to thio­phene derivatives, see: Molvi *et al.* (2007 ▶); Rai *et al.* (2008 ▶). For applications of 2-amino­thio­phene derivatives, see: Puterová *et al.* (2010 ▶); Cannito *et al.* (1990 ▶); Nikolakopoulos *et al.* (2006 ▶). For biological and industrial applications of Schiff bases, see: Desai *et al.* (2001 ▶); Singh & Dash (1988 ▶); Aydogan *et al.* (2001 ▶); Taggi *et al.* (2002 ▶). For a related structure, see: Fun *et al.* (2012 ▶). For standard bond lengths, see: Allen *et al.* (1987 ▶). 
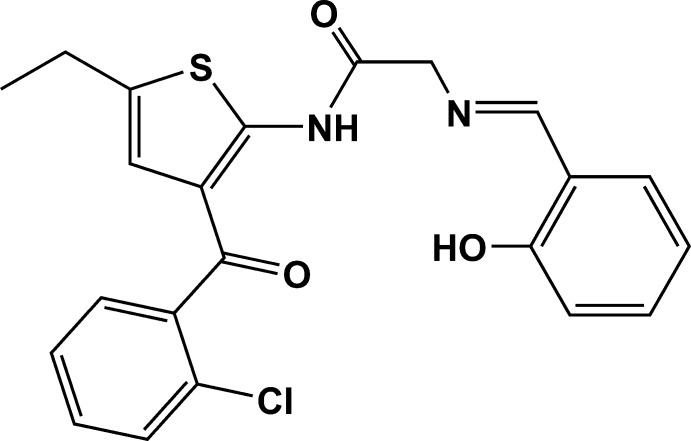


## Experimental   

### Crystal data  

C_22_H_19_ClN_2_O_3_S
*M* *_r_* = 426.90
Monoclinic, 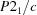

*a* = 9.7888 (2) Å
*b* = 16.9476 (3) Å
*c* = 12.2863 (3) Å
β = 90.6654 (19)°
*V* = 2038.11 (7) Å^3^
*Z* = 4
Cu *K*α radiation
μ = 2.84 mm^−1^
*T* = 173 K
0.32 × 0.28 × 0.18 mm

### Data collection  

Agilent Xcalibur Eos Gemini diffractometer
Absorption correction: multi-scan (*CrysAlis PRO*; Agilent, 2012 ▶) *T*_min_ = 0.802, *T*_max_ = 1.000
14124 measured reflections
3909 independent reflections
3459 reflections with *I* > 2σ(*I*)
*R*_int_ = 0.028

### Refinement  

*R*[*F* ^2^ > 2σ(*F* ^2^)] = 0.037
*wR*(*F* ^2^) = 0.102
*S* = 1.02
3909 reflections
264 parameters
H-atom parameters constrained
Δρ_max_ = 0.42 e Å^−3^
Δρ_min_ = −0.29 e Å^−3^

### 

Data collection: *CrysAlis PRO* (Agilent, 2012 ▶); cell refinement: *CrysAlis PRO*; data reduction: *CrysAlis RED* (Agilent, 2012 ▶); program(s) used to solve structure: *SUPERFLIP* (Palatinus & Chapuis, 2007 ▶); program(s) used to refine structure: *SHELXL2012* (Sheldrick, 2008 ▶); molecular graphics: *OLEX2* (Dolomanov *et al.*, 2009 ▶); software used to prepare material for publication: *OLEX2*.

## Supplementary Material

Crystal structure: contains datablock(s) I. DOI: 10.1107/S1600536814018224/zs2311sup1.cif

Structure factors: contains datablock(s) I. DOI: 10.1107/S1600536814018224/zs2311Isup2.hkl

Supporting information file. DOI: 10.1107/S1600536814018224/zs2311Isup3.cml

**Fig. 1.**
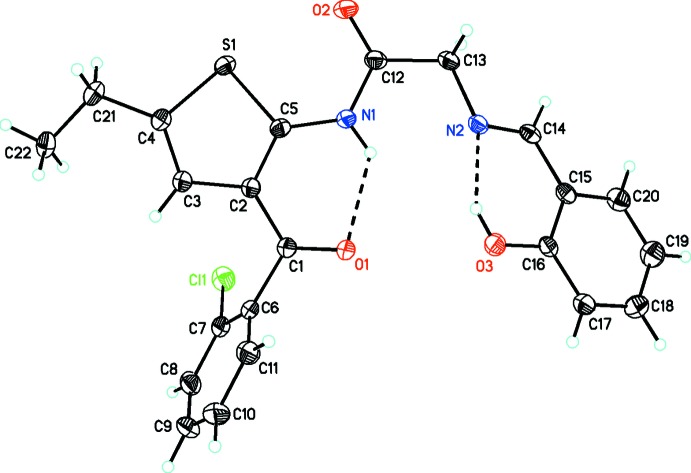
ORTEP drawing of C22H19N2O3SCl showing the labeling scheme of the molecule with 30% probability displacement ellipsoids. Dashed lines inidicate O—H···N and N—H···O intramolecular hydrogen bonds.

22 19 2 3 . DOI: 10.1107/S1600536814018224/zs2311fig1.tif
ORTEP drawing of C_22_H_19_N_2_O_3_SCl showing the labeling scheme of the mol­ecule with 30% probability displacement ellipsoids. Dashed lines inidicate O—H⋯N and N—H⋯O intra­molecular hydrogen bonds.

**Fig. 2.**
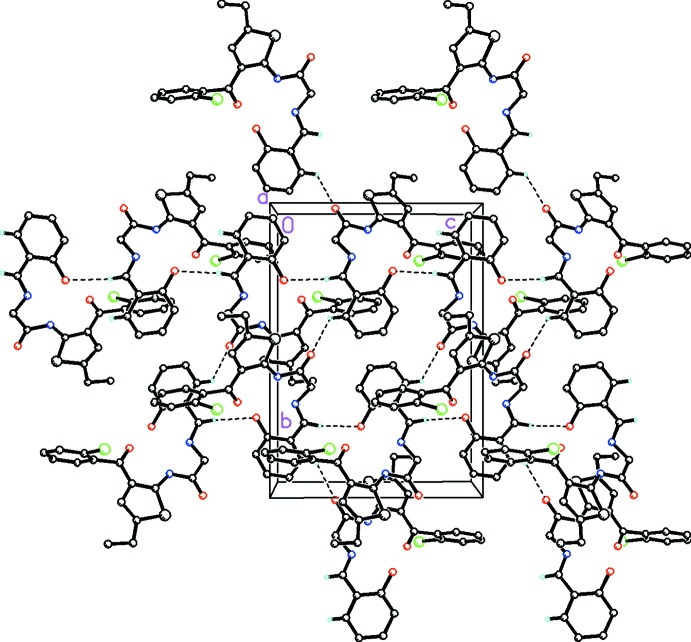
Molecular packing for C22H19ClN2O3S in the unit cell viewed along the a axis. Dashed lines indicate weak C—H···O intermolecular interactions which interlink the molecules forming chains along the b and c axes. H atoms not involved in hydrogen- bonding have been removed for clarity.

22 19 2 3 a b c . DOI: 10.1107/S1600536814018224/zs2311fig2.tif
Mol­ecular packing for C_22_H_19_ClN_2_O_3_S in the unit cell viewed along the *a* axis. Dashed lines indicate weak C—H⋯O inter­molecular inter­actions which inter­link the mol­ecules forming chains along the *b* and *c* axes. H atoms not involved in hydrogen- bonding have been removed for clarity.

CCDC reference: 1018596

Additional supporting information:  crystallographic information; 3D view; checkCIF report

## Figures and Tables

**Table 1:** Hydrogen-bond geometry (Å, °)

*D*—H⋯*A*	*D*—H	H⋯*A*	*D*⋯*A*	*D*—H⋯*A*
O3—H3⋯N2	0.82	1.94	2.6611 (19)	146
N1—H1⋯O1	0.86	2.08	2.7177 (19)	130
C14—H14⋯O3^i^	0.93	2.56	3.405 (2)	152
C20—H20⋯O2^ii^	0.93	2.57	3.209 (2)	126

Symmetry codes: (i) 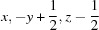; (ii) 
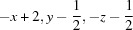
.

## supplementary crystallographic information

### S1. Comment

Thiophene derivatives have been reported to exhibit a broad spectrum of biological properties such as anti-inflammatory, analgesic, anti-depressant, anti-microbial and anti-convulsant activities (Molvi *et al.*, 2007; Rai *et al.*, 2008). 2-Aminothiophene derivatives have been used in a number of applications in pesticides, dyes and pharmaceuticals. Reviews on the synthesis and properties of these compounds have been reported (Puterová *et al.*, 2010). Substituted 2-aminothiophenes are active as allosteric enhancers at the human A1 adenosine receptor (Cannito *et al.*,1990; Nikolakopoulos *et al.*, 2006). Schiff base compounds are an important class of compounds both synthetically and biologically. These compounds show biological properties including anti-bacterial, anti-fungal, anti-cancer and herbicidal activities (Desai *et al.*, 2001; Singh & Dash, 1988). Furthermore, Schiff bases are utilized as starting materials in the synthesis of compounds of industrial (Aydogan *et al.*, 2001) and biological interest such as β-lactams (Taggi *et al.*, 2002). The crystal and molecular structure of the reactant 2-aminothiophene has been previously reported by our group (Fun *et al.*, 2012). In view of the importance of 2-aminothiphenes and Schiff bases, we report herein the crystal structure of the Schiff base of the previously reported 2-aminothiophene, the title compound, C_22_H_19_ClN_2_O_3_S, (I).

In (I), the dihedral angle between the mean planes of the thiophene ring and the chlorophenyl and hydroxyphenyl rings is 70.1 (1)° and 40.2 (4)°, respectively (Fig. 1). The two phenyl rings are twisted with respect to each other by 88.9 (3)°. The imine bond lies in an *E* conformation. Bond lengths are in normal ranges (Allen *et al.*, 1987). Intramolecular O3—H3···N2 and N1—H1···O1 hydrogen bonds each generate *S*(6) ring motifs (Table 1). In the crystal, weak C14–H···O3 intermolecular interactions link the molecules forming infinite one-dimensional linear chains along the *c* axis while weak C20—H···O2 intermolecular interactions form zig-zag chains along the *b* axis, generating a two- dimensional network structure lying parallel to (100) (Fig. 2).

### S2. Experimental

To a solution of 2-amino-N-[3-(2-chloro-benzoyl)-5-ethyl-thiophen-2-yl]- acetamide (200 mg, 0.62 mmol) in 10 ml of methanol an equimolar amount of salicylaldehyde (76 mg, 0.62 mmol) was added dropwise with constant stirring. The mixture was refluxed for 4 hours producing a pale yellow precipitate. The reaction completion was confirmed by thin layer chromatography. The precipitate was filtered and dried at room temperature overnight. The solid was recrystallized using dichloromethane and the crystals were used as such for X-ray diffraction studies.

### S3. Refinement

All of the H atoms were placed in their calculated positions and then refined using the riding model with atom—H lengths of 0.93Å (CH); 0.97Å (CH_2_); 0.96Å (CH_3_); 0.82Å (OH) or 0.86Å (NH). Isotropic displacement parameters for these atoms were set to 1.2 (CH, CH_2_, NH) or 1.5 (CH_3_, OH) times *U*_eq_ of the parent atom. Idealised Me and OH were refined as rotating groups.

### Crystal data

**Table:**

C_22_H_19_ClN_2_O_3_S	*F*(000) = 888
*M**_r_* = 426.90	*D*_x_ = 1.391 Mg m^−^^3^
Monoclinic, *P*2_1_/*c*	Cu *K*α radiation, λ = 1.54184 Å
*a* = 9.7888 (2) Å	Cell parameters from 6410 reflections
*b* = 16.9476 (3) Å	θ = 4.4–71.5°
*c* = 12.2863 (3) Å	µ = 2.84 mm^−^^1^
β = 90.6654 (19)°	*T* = 173 K
*V* = 2038.11 (7) Å^3^	Irregular, pale yellow
*Z* = 4	0.32 × 0.28 × 0.18 mm

### Data collection

**Table:**

Agilent Xcalibur Eos Gemini diffractometer	3909 independent reflections
Radiation source: Enhance (Cu) X-ray Source	3459 reflections with *I* > 2σ(*I*)
Detector resolution: 16.0416 pixels mm^-1^	*R*_int_ = 0.028
ω scans	θ_max_ = 71.4°, θ_min_ = 4.5°
Absorption correction: multi-scan (*CrysAlis PRO*; Agilent, 2012)	*h* = −12→10
*T*_min_ = 0.802, *T*_max_ = 1.000	*k* = −15→20
14124 measured reflections	*l* = −15→14

### Refinement

**Table:**

Refinement on *F*^2^	Primary atom site location: structure-invariant direct methods
Least-squares matrix: full	Hydrogen site location: inferred from neighbouring sites
*R*[*F*^2^ > 2σ(*F*^2^)] = 0.037	H-atom parameters constrained
*wR*(*F*^2^) = 0.102	*w* = 1/[σ^2^(*F*_o_^2^) + (0.0595*P*)^2^ + 0.7231*P*] where *P* = (*F*_o_^2^ + 2*F*_c_^2^)/3
*S* = 1.02	(Δ/σ)_max_ = 0.001
3909 reflections	Δρ_max_ = 0.42 e Å^−^^3^
264 parameters	Δρ_min_ = −0.29 e Å^−^^3^
0 restraints	

### Special details

**Table:**

Experimental. 1H NMR (400 MHz, CDCl3): δ 12.39 (s, 1H), 12.18 (s, 1H), 8.51 (s, 1H), 7.45-7.28 (m, 6H), 7.06 (d, J = 8.4 Hz, 1H), 6.92 (t, J = 7.6 Hz, 1H), 6.38 (d, J = 1.2 Hz, 1H), 4.61 (s, 2H), 2.70 (q, J = 7.6 Hz, 2H), 1.27-1.23 (m, 3H).13C NMR (400 MHz, CDCl3): δ 191.0, 169.4, 166.8, 160.7, 148.4, 139.4, 137.4, 133.3, 132.3, 130.7, 130.5, 129.9, 128.3, 126.6, 121.3, 121.2, 121.1, 119.0, 118.4, 117.4, 62.8, 22.8, 15.5. MS: m/z = 426.91 (Calculated), m/z = 426.94 [M]^+^ (found).
Geometry. All esds (except the esd in the dihedral angle between two l.s. planes) are estimated using the full covariance matrix. The cell esds are taken into account individually in the estimation of esds in distances, angles and torsion angles; correlations between esds in cell parameters are only used when they are defined by crystal symmetry. An approximate (isotropic) treatment of cell esds is used for estimating esds involving l.s. planes.

### Fractional atomic coordinates and isotropic or equivalent isotropic displacement parameters (Å^2^)

**Table:**

	*x*	*y*	*z*	*U*_iso_*/*U*_eq_	
Cl1	0.51629 (5)	0.31401 (3)	0.21556 (4)	0.04758 (15)	
S1	0.72524 (4)	0.53992 (2)	−0.03408 (3)	0.03034 (13)	
O1	0.90220 (15)	0.32692 (8)	0.14607 (11)	0.0450 (4)	
O2	0.89582 (15)	0.48587 (9)	−0.19425 (12)	0.0488 (4)	
O3	1.20084 (14)	0.26518 (8)	0.07197 (10)	0.0389 (3)	
H3	1.1619	0.2959	0.0304	0.058*	
N1	0.89927 (14)	0.41330 (9)	−0.04031 (11)	0.0298 (3)	
H1	0.9353	0.3731	−0.0084	0.036*	
N2	1.09746 (14)	0.31536 (9)	−0.11697 (11)	0.0296 (3)	
C1	0.81400 (18)	0.37146 (11)	0.18143 (14)	0.0337 (4)	
C2	0.75997 (17)	0.43810 (10)	0.12005 (14)	0.0301 (4)	
C3	0.65870 (17)	0.49297 (10)	0.15660 (14)	0.0312 (4)	
H3A	0.6172	0.4893	0.2241	0.037*	
C4	0.62912 (17)	0.55044 (10)	0.08380 (14)	0.0303 (4)	
C5	0.80425 (17)	0.45664 (10)	0.01659 (14)	0.0277 (3)	
C6	0.76476 (19)	0.35931 (11)	0.29613 (14)	0.0347 (4)	
C7	0.6345 (2)	0.33302 (10)	0.31984 (15)	0.0361 (4)	
C8	0.5958 (2)	0.31975 (11)	0.42645 (17)	0.0459 (5)	
H8	0.5078	0.3025	0.4416	0.055*	
C9	0.6891 (3)	0.33232 (14)	0.50999 (17)	0.0542 (6)	
H9	0.6642	0.3226	0.5816	0.065*	
C10	0.8190 (3)	0.35918 (16)	0.48780 (18)	0.0579 (6)	
H10	0.8811	0.3679	0.5443	0.069*	
C11	0.8567 (2)	0.37316 (14)	0.38070 (17)	0.0479 (5)	
H11	0.9438	0.3919	0.3658	0.058*	
C12	0.93954 (17)	0.42990 (11)	−0.14303 (14)	0.0325 (4)	
C13	1.04302 (19)	0.37442 (12)	−0.19171 (14)	0.0366 (4)	
H13A	1.0007	0.3476	−0.2531	0.044*	
H13B	1.1184	0.4054	−0.2193	0.044*	
C14	1.13375 (17)	0.24926 (10)	−0.15744 (13)	0.0288 (3)	
H14	1.1191	0.2404	−0.2314	0.035*	
C15	1.19709 (17)	0.18724 (10)	−0.09226 (14)	0.0285 (3)	
C16	1.22675 (17)	0.19676 (10)	0.01912 (13)	0.0289 (3)	
C17	1.28505 (19)	0.13503 (12)	0.07805 (15)	0.0376 (4)	
H17	1.3040	0.1412	0.1519	0.045*	
C18	1.3150 (2)	0.06443 (12)	0.02676 (18)	0.0461 (5)	
H18	1.3542	0.0234	0.0665	0.055*	
C19	1.2869 (2)	0.05440 (12)	−0.08316 (19)	0.0483 (5)	
H19	1.3071	0.0068	−0.1171	0.058*	
C20	1.2291 (2)	0.11509 (11)	−0.14174 (15)	0.0387 (4)	
H20	1.2109	0.1082	−0.2156	0.046*	
C21	0.52840 (19)	0.61717 (10)	0.09042 (17)	0.0373 (4)	
H21A	0.4703	0.6163	0.0259	0.045*	
H21B	0.5780	0.6667	0.0907	0.045*	
C22	0.43888 (19)	0.61370 (11)	0.19068 (16)	0.0387 (4)	
H22A	0.4948	0.6193	0.2549	0.058*	
H22B	0.3922	0.5639	0.1927	0.058*	
H22C	0.3731	0.6557	0.1877	0.058*	

### Atomic displacement parameters (Å^2^)

**Table:**

	*U*^11^	*U*^22^	*U*^33^	*U*^12^	*U*^13^	*U*^23^
Cl1	0.0494 (3)	0.0435 (3)	0.0499 (3)	−0.0040 (2)	0.0038 (2)	−0.0039 (2)
S1	0.0306 (2)	0.0277 (2)	0.0327 (2)	0.00170 (15)	−0.00142 (16)	0.00588 (15)
O1	0.0500 (8)	0.0504 (8)	0.0350 (7)	0.0261 (6)	0.0130 (6)	0.0114 (6)
O2	0.0476 (8)	0.0570 (9)	0.0421 (8)	0.0170 (7)	0.0090 (6)	0.0233 (7)
O3	0.0495 (8)	0.0438 (7)	0.0234 (6)	0.0149 (6)	−0.0037 (5)	−0.0071 (5)
N1	0.0283 (7)	0.0330 (7)	0.0281 (7)	0.0051 (6)	0.0024 (5)	0.0060 (6)
N2	0.0289 (7)	0.0377 (8)	0.0222 (7)	0.0016 (6)	0.0019 (5)	0.0006 (6)
C1	0.0326 (9)	0.0373 (9)	0.0313 (9)	0.0087 (7)	0.0046 (7)	0.0046 (7)
C2	0.0282 (8)	0.0329 (9)	0.0292 (8)	0.0048 (7)	0.0022 (6)	0.0026 (7)
C3	0.0300 (8)	0.0319 (8)	0.0318 (9)	0.0039 (7)	0.0013 (7)	0.0003 (7)
C4	0.0290 (8)	0.0273 (8)	0.0346 (9)	0.0004 (6)	−0.0021 (7)	−0.0009 (7)
C5	0.0263 (8)	0.0279 (8)	0.0287 (8)	−0.0002 (6)	−0.0025 (6)	0.0032 (6)
C6	0.0397 (9)	0.0348 (9)	0.0298 (9)	0.0162 (7)	0.0055 (7)	0.0061 (7)
C7	0.0450 (10)	0.0277 (8)	0.0358 (9)	0.0076 (7)	0.0076 (8)	0.0017 (7)
C8	0.0620 (13)	0.0324 (9)	0.0438 (11)	0.0026 (9)	0.0207 (10)	0.0019 (8)
C9	0.0804 (17)	0.0517 (13)	0.0308 (10)	0.0159 (11)	0.0193 (10)	0.0085 (9)
C10	0.0647 (15)	0.0753 (16)	0.0335 (11)	0.0231 (12)	−0.0056 (10)	0.0055 (10)
C11	0.0395 (10)	0.0667 (14)	0.0376 (10)	0.0179 (9)	0.0027 (8)	0.0057 (9)
C12	0.0276 (8)	0.0414 (10)	0.0284 (8)	0.0007 (7)	0.0002 (6)	0.0081 (7)
C13	0.0366 (9)	0.0484 (10)	0.0248 (8)	0.0064 (8)	0.0062 (7)	0.0085 (7)
C14	0.0292 (8)	0.0382 (9)	0.0190 (7)	−0.0056 (7)	0.0013 (6)	−0.0014 (6)
C15	0.0268 (8)	0.0330 (8)	0.0259 (8)	−0.0054 (6)	0.0029 (6)	−0.0027 (6)
C16	0.0261 (8)	0.0356 (9)	0.0251 (8)	0.0002 (6)	0.0044 (6)	−0.0024 (7)
C17	0.0362 (9)	0.0472 (10)	0.0296 (9)	0.0042 (8)	0.0031 (7)	0.0032 (8)
C18	0.0521 (12)	0.0377 (10)	0.0486 (12)	0.0065 (9)	0.0031 (9)	0.0087 (9)
C19	0.0615 (13)	0.0306 (9)	0.0529 (12)	0.0017 (9)	0.0053 (10)	−0.0071 (9)
C20	0.0465 (10)	0.0366 (10)	0.0330 (9)	−0.0048 (8)	0.0006 (8)	−0.0080 (7)
C21	0.0339 (9)	0.0270 (8)	0.0511 (11)	0.0046 (7)	−0.0016 (8)	0.0019 (8)
C22	0.0364 (9)	0.0349 (9)	0.0447 (10)	0.0069 (7)	−0.0049 (8)	−0.0103 (8)

### Geometric parameters (Å, º)

**Table:**

Cl1—C7	1.746 (2)	C9—C10	1.381 (4)
S1—C4	1.7453 (18)	C10—H10	0.9300
S1—C5	1.7223 (16)	C10—C11	1.391 (3)
O1—C1	1.230 (2)	C11—H11	0.9300
O2—C12	1.214 (2)	C12—C13	1.511 (3)
O3—H3	0.8200	C13—H13A	0.9700
O3—C16	1.354 (2)	C13—H13B	0.9700
N1—H1	0.8600	C14—H14	0.9300
N1—C5	1.382 (2)	C14—C15	1.456 (2)
N1—C12	1.356 (2)	C15—C16	1.405 (2)
N2—C13	1.455 (2)	C15—C20	1.403 (2)
N2—C14	1.278 (2)	C16—C17	1.391 (3)
C1—C2	1.454 (2)	C17—H17	0.9300
C1—C6	1.509 (2)	C17—C18	1.385 (3)
C2—C3	1.435 (2)	C18—H18	0.9300
C2—C5	1.384 (2)	C18—C19	1.386 (3)
C3—H3A	0.9300	C19—H19	0.9300
C3—C4	1.351 (2)	C19—C20	1.373 (3)
C4—C21	1.503 (2)	C20—H20	0.9300
C6—C7	1.385 (3)	C21—H21A	0.9700
C6—C11	1.387 (3)	C21—H21B	0.9700
C7—C8	1.386 (3)	C21—C22	1.521 (3)
C8—H8	0.9300	C22—H22A	0.9600
C8—C9	1.382 (4)	C22—H22B	0.9600
C9—H9	0.9300	C22—H22C	0.9600
			
C5—S1—C4	91.60 (8)	N1—C12—C13	116.29 (15)
C16—O3—H3	109.5	N2—C13—C12	114.87 (14)
C5—N1—H1	117.8	N2—C13—H13A	108.5
C12—N1—H1	117.8	N2—C13—H13B	108.5
C12—N1—C5	124.42 (15)	C12—C13—H13A	108.5
C14—N2—C13	117.32 (14)	C12—C13—H13B	108.5
O1—C1—C2	123.10 (16)	H13A—C13—H13B	107.5
O1—C1—C6	118.64 (15)	N2—C14—H14	118.7
C2—C1—C6	118.17 (14)	N2—C14—C15	122.52 (15)
C3—C2—C1	126.12 (15)	C15—C14—H14	118.7
C5—C2—C1	122.47 (15)	C16—C15—C14	122.33 (15)
C5—C2—C3	111.41 (15)	C20—C15—C14	119.14 (15)
C2—C3—H3A	123.1	C20—C15—C16	118.53 (16)
C4—C3—C2	113.79 (16)	O3—C16—C15	121.85 (15)
C4—C3—H3A	123.1	O3—C16—C17	118.19 (15)
C3—C4—S1	111.23 (13)	C17—C16—C15	119.97 (16)
C3—C4—C21	129.94 (17)	C16—C17—H17	120.0
C21—C4—S1	118.82 (14)	C18—C17—C16	120.03 (17)
N1—C5—S1	123.71 (13)	C18—C17—H17	120.0
N1—C5—C2	124.32 (15)	C17—C18—H18	119.7
C2—C5—S1	111.96 (13)	C17—C18—C19	120.58 (19)
C7—C6—C1	123.05 (17)	C19—C18—H18	119.7
C7—C6—C11	119.20 (17)	C18—C19—H19	120.2
C11—C6—C1	117.73 (17)	C20—C19—C18	119.65 (18)
C6—C7—Cl1	120.58 (14)	C20—C19—H19	120.2
C6—C7—C8	120.9 (2)	C15—C20—H20	119.4
C8—C7—Cl1	118.52 (17)	C19—C20—C15	121.23 (18)
C7—C8—H8	120.3	C19—C20—H20	119.4
C9—C8—C7	119.4 (2)	C4—C21—H21A	108.9
C9—C8—H8	120.3	C4—C21—H21B	108.9
C8—C9—H9	119.8	C4—C21—C22	113.53 (16)
C10—C9—C8	120.41 (19)	H21A—C21—H21B	107.7
C10—C9—H9	119.8	C22—C21—H21A	108.9
C9—C10—H10	120.1	C22—C21—H21B	108.9
C9—C10—C11	119.9 (2)	C21—C22—H22A	109.5
C11—C10—H10	120.1	C21—C22—H22B	109.5
C6—C11—C10	120.2 (2)	C21—C22—H22C	109.5
C6—C11—H11	119.9	H22A—C22—H22B	109.5
C10—C11—H11	119.9	H22A—C22—H22C	109.5
O2—C12—N1	122.69 (17)	H22B—C22—H22C	109.5
O2—C12—C13	121.02 (16)		
			
Cl1—C7—C8—C9	178.20 (15)	C5—S1—C4—C21	−178.57 (14)
S1—C4—C21—C22	172.69 (13)	C5—N1—C12—O2	0.9 (3)
O1—C1—C2—C3	−179.10 (19)	C5—N1—C12—C13	−179.01 (16)
O1—C1—C2—C5	−0.1 (3)	C5—C2—C3—C4	−0.4 (2)
O1—C1—C6—C7	−111.3 (2)	C6—C1—C2—C3	−2.5 (3)
O1—C1—C6—C11	66.8 (3)	C6—C1—C2—C5	176.43 (17)
O2—C12—C13—N2	173.46 (17)	C6—C7—C8—C9	−0.6 (3)
O3—C16—C17—C18	−179.09 (18)	C7—C6—C11—C10	1.3 (3)
N1—C12—C13—N2	−6.6 (2)	C7—C8—C9—C10	1.1 (3)
N2—C14—C15—C16	−2.1 (2)	C8—C9—C10—C11	−0.5 (4)
N2—C14—C15—C20	177.49 (16)	C9—C10—C11—C6	−0.8 (4)
C1—C2—C3—C4	178.67 (17)	C11—C6—C7—Cl1	−179.40 (15)
C1—C2—C5—S1	−178.41 (14)	C11—C6—C7—C8	−0.7 (3)
C1—C2—C5—N1	2.3 (3)	C12—N1—C5—S1	−1.4 (2)
C1—C6—C7—Cl1	−1.3 (2)	C12—N1—C5—C2	177.77 (17)
C1—C6—C7—C8	177.39 (16)	C13—N2—C14—C15	176.28 (15)
C1—C6—C11—C10	−176.8 (2)	C14—N2—C13—C12	149.92 (16)
C2—C1—C6—C7	72.0 (2)	C14—C15—C16—O3	−1.5 (3)
C2—C1—C6—C11	−110.0 (2)	C14—C15—C16—C17	178.81 (15)
C2—C3—C4—S1	−0.1 (2)	C14—C15—C20—C19	−178.92 (18)
C2—C3—C4—C21	178.74 (17)	C15—C16—C17—C18	0.6 (3)
C3—C2—C5—S1	0.69 (19)	C16—C15—C20—C19	0.7 (3)
C3—C2—C5—N1	−178.61 (16)	C16—C17—C18—C19	−0.2 (3)
C3—C4—C21—C22	−6.1 (3)	C17—C18—C19—C20	0.1 (3)
C4—S1—C5—N1	178.67 (15)	C18—C19—C20—C15	−0.4 (3)
C4—S1—C5—C2	−0.63 (14)	C20—C15—C16—O3	178.86 (16)
C5—S1—C4—C3	0.40 (14)	C20—C15—C16—C17	−0.8 (2)

### Hydrogen-bond geometry (Å, º)

**Table:**

*D*—H···*A*	*D*—H	H···*A*	*D*···*A*	*D*—H···*A*
O3—H3···N2	0.82	1.94	2.6611 (19)	146
N1—H1···O1	0.86	2.08	2.7177 (19)	130
C14—H14···O3^i^	0.93	2.56	3.405 (2)	152
C20—H20···O2^ii^	0.93	2.57	3.209 (2)	126

Symmetry codes: (i) *x*, −*y*+1/2, *z*−1/2; (ii) −*x*+2, *y*−1/2, −*z*−1/2.
