# Supplementary figures and images for: Crystal structure of N-[3-(2-chloro­benzo­yl)-5-ethyl­thio­phen-2-yl]-2-[(E)-(2-hy­droxy­benzyl­idene)amino]­acetamide
Source: Acta Crystallogr Sect E Struct Rep Online. 2014 Aug 16;70(Pt 9):o1011–2. doi: 10.1107/S1600536814018224 (PMC4186110; doi:10.1107/S1600536814018224)

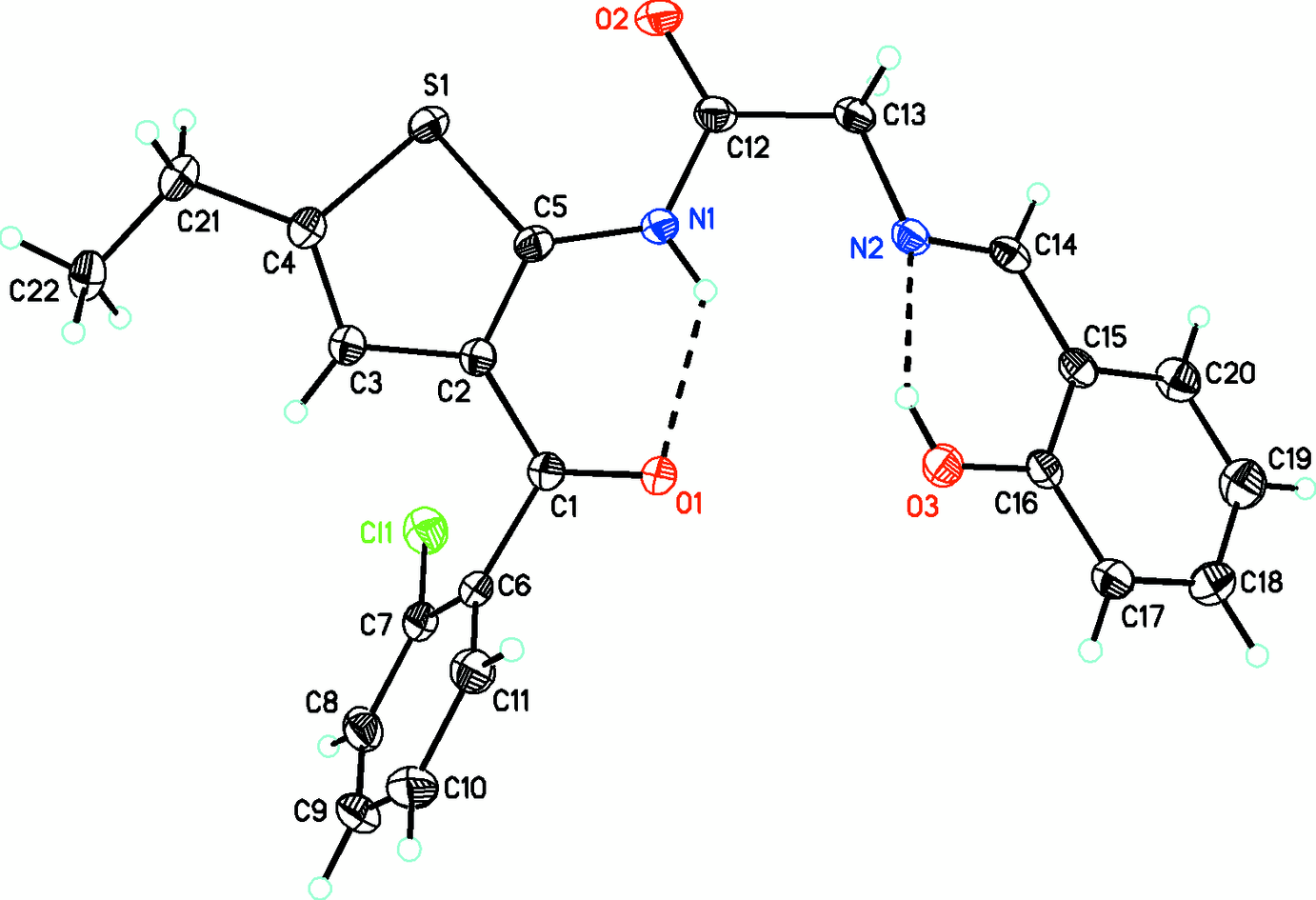

Supplement: Supplementary file 4 [file e-70-o1011-fig1.tif]

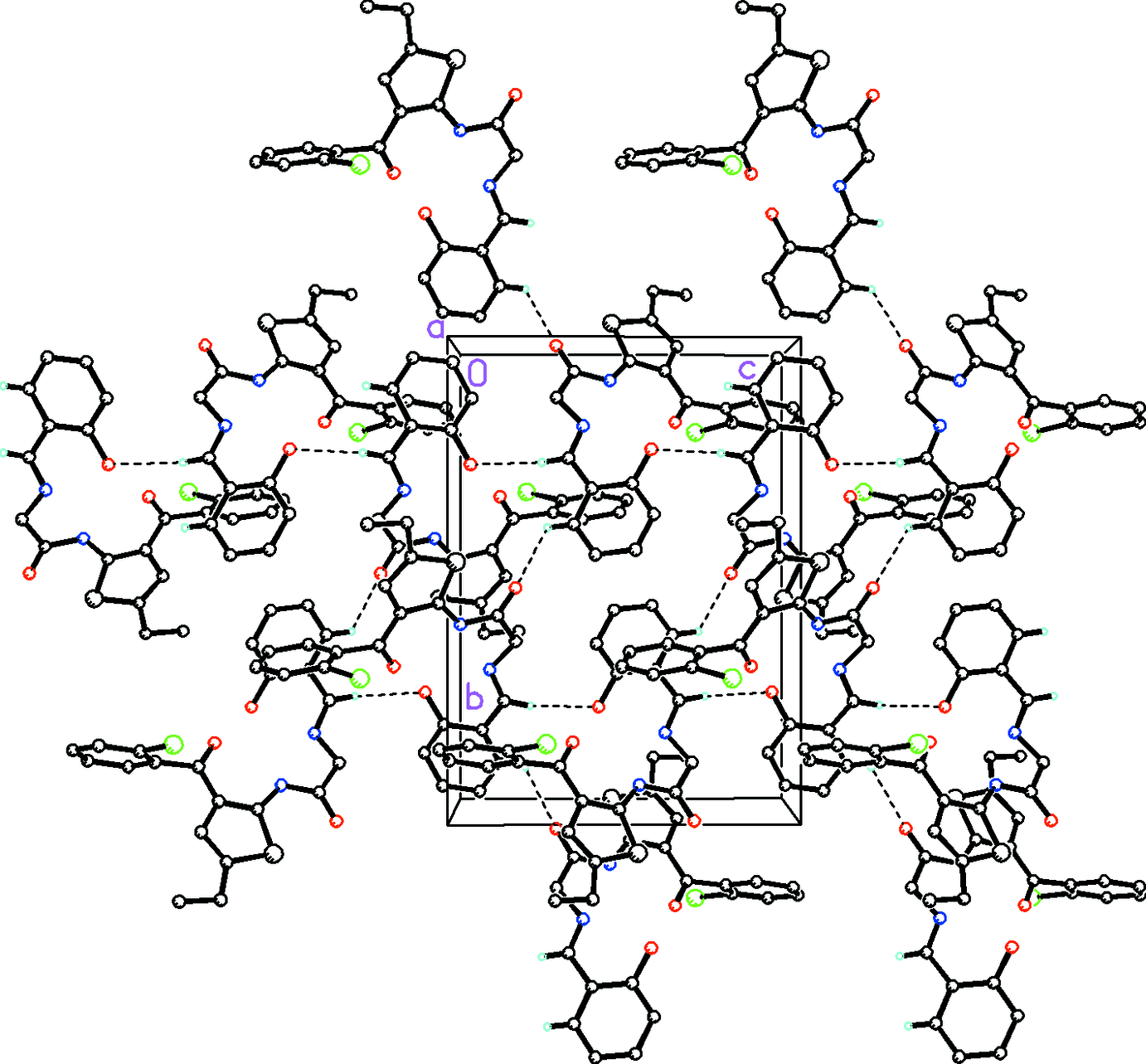

Supplement: Supplementary file 5 [file e-70-o1011-fig2.tif]
